# An Overview on Fecal Branched Short-Chain Fatty Acids Along Human Life and as Related With Body Mass Index: Associated Dietary and Anthropometric Factors

**DOI:** 10.3389/fmicb.2020.00973

**Published:** 2020-05-27

**Authors:** David Rios-Covian, Sonia González, Alicja M. Nogacka, Silvia Arboleya, Nuria Salazar, Miguel Gueimonde, Clara G. de los Reyes-Gavilán

**Affiliations:** ^1^Department of Microbiology and Biochemistry of Dairy Products, Instituto de Productos Lácteos de Asturias, Consejo Superior de Investigaciones Científicas (IPLA-CSIC), Villaviciosa, Spain; ^2^Department of Functional Biology, University of Oviedo, Oviedo, Spain; ^3^Diet, Microbiota and Health Group, Instituto de Investigación Sanitaria del Principado de Asturias (DIMISA-ISPA), Oviedo, Spain

**Keywords:** short-chain fatty acids, branched short-chain fatty acids, isovaleric acid, isobutyric acid, BMI, age, diet

## Abstract

Short-chain fatty acids (SCFA) are the main bacterial products of the catabolism of carbohydrates and proteins in the gut, and their role is essential in host–microbiota interactions. Acetic, propionic, and butyric acids are the major SCFA produced in the gut, and they have been extensively studied. In contrast, branched short-chain fatty acids (BCFA), mainly isovaleric and isobutyric acids, are produced in less amounts and their fecal levels in different human groups, intestinal microbial producing populations, and influence on health are insufficiently known. They have been proposed as markers of protein fermentation, which leads to the concomitant production of other fermentation products that can be harmful for the colon epithelium. In this context, the aim of this study was to shed light into the production of BCFA by the human intestinal microbiota, as related to age, body mass index (BMI), and diet. Fecal levels of the different SCFA were analyzed by gas chromatography in 232 healthy individuals with ages between 3 months and 95 years, and BMI in adults ranging from 19 to 54. Dietary assessments in adults were obtained through a food frequency questionnaire (FFQ). Molar proportions of BCFA in feces were strongly and positively related with aging. However, not a significant relationship was obtained between BCFA and BMI. A negative correlation was found between the consumption of dietary insoluble fiber and fecal levels of BCFA. More studies are needed for improving our understanding on the relationship of BCFA production profile with the intestinal microbiota composition and human health.

## Introduction

The human gut is inhabited by billions of microorganisms that accomplish several essential functions for host health (Lozupone et al., 2012). Among them, the fermentation of undigested dietary components is of paramount importance for the physiology and metabolism of the host. The subsequent microbial released metabolites have a key role in the interplay between bacterial producers and other gut inhabitants as well as with the host cells (Macfarlane and Macfarlane, 2012). Acetic, propionic, and butyric acids are the major catabolic end-products from the fermentation by the intestinal microbiota of dietary undigested carbohydrates and proteins, and they are commonly known as short-chain fatty acids (SCFA). Until present, caproic and valeric acids in the gut have been associated with diet and have not been considered as SCFA strictly produced by the microbiota. However, recent researches have described the ability of some species from the gut microbiota to produce caproic acid from lactate by cross-feeding (Zhu et al., 2017) whereas the production of valeric acid by the intestinal microbiota has also been suggested (McDonald et al., 2018). During recent years, acetic, propionic, and butyric acids have received a considerable deal of attention, their production pathways have been described, and fecal levels have been determined in individuals from different geographical locations, along life, and in various disease states, as related with their effects on host health (Ríos-Covián et al., 2016). Apart from these major SCFA, the intestinal microbiota also produces considerably lower amounts of isobutyric, isovaleric, and 2-methylbutyric acids, commonly known as branched short-chain fatty acids (BCFA).

It is known that BCFA are mainly produced during fermentation of branched chain amino acids (valine, leucine, and isoleucine) by the intestinal microbiota. In the human intestine, the fermentation of branched chain amino acids is carried out mainly by genera *Bacteroides* and *Clostridium* (Smith and Macfarlane, 1998; Aguirre et al., 2016) and the levels of BCFA increase from the proximal colon to the distal colon and feces (Macfarlane et al., 1992).

*In vitro* studies using protein-fermenting bacteria have demonstrated BCFA production when the microorganisms were grown with peptides as the main carbon source at low pH 6.8; however, the presence of starch at pH (5.5) reduced the formation of BCFA in these cultures (Smith and Macfarlane, 1998). Evidences also indicate that high-protein and low-complex carbohydrate diets, like western diet, result in higher concentrations of BCFA in a validated *in vitro* gut model (Aguirre et al., 2016), which has been further corroborated in some dietary interventions carried out in animals and humans (Pieper et al., 2012; Hald et al., 2016). In general, dietary supplementation with complex carbohydrates, able to reach the colon, results in a decrease in fecal levels of BCFA (François et al., 2014) whereas protein supplementation drifts into higher production of these compounds by the intestinal microbiota (Geypens et al., 1997; Russell et al., 2011). To date, all the literature regarding the relationship between protein intake and BCFA production has been based on dietary interventions with supplements, with no observational studies currently available comparing the BCFA production in different population groups with different intakes of protein at baseline. In addition, BCFA have attracted considerably less attention than major SCFA (acetate, propionate, and butyrate), in spite that they might be playing important roles in the gut environment and may constitute potential markers of the microbial metabolism taking place in the gut. BCFA have been proposed as markers of colonic protein fermentation (Macfarlane et al., 1992), a process that leads to the concomitant production of other protein fermentation products such as ammonia, phenol, p-cresol, or biogenic amines, molecules that can cause cell damage on the intestinal environment (Aguirre et al., 2016). Some authors have reported that isobutyrate stimulates colonic Na^+^ absorption (Blachier et al., 2007), high levels of isovalerate in feces have been related with human depression and cortisol levels (Szczesniak et al., 2016), and, more recently, a role of BCFA on the regulation of glucose and lipid metabolism has been also suggested (Heimann et al., 2016). However, to date and despite the knowledge on their biosynthetic pathways, no integrated information exists on fecal levels of BCFA along life and in different health status.

In this study, we aimed at assessing the variations in fecal levels of BCFA according to age, from lactating infants to the elderly, and on the basis of body mass index (BMI). Moreover, the possible association with general dietary habits of the adult population was also evaluated. To the best of our knowledge, this is the first report providing information on BCFA in human feces throughout life.

## Materials and Methods

### Participants

The study sample included 232 subjects, 81 males and 151 females, from 3 months to 95 years of age, and with BMI values for adults ranging from 19.02 to 54.50 (Tables 1, 2). All volunteers were recruited in Asturias region (Northern Spain) in the frame of different studies carried out by our research group (Arboleya et al., 2012, 2016; Salazar et al., 2013; Fernandez-Navarro et al., 2017). In addition to the previously available subjects, 12 males and 26 females with ages between 34 and 59 years and BMI between 20 and 54 were specifically recruited and added to the study database. All newborns were recruited at 3 months of age. For descriptive purposes, the adult sample was categorized in age quartiles on the basis of sample distribution and in BMI following the BMI chart diving in normal-weight (18.5–24.9), overweight (25–29.9), obese (30–39.9), and extremely obese (≥40) subjects. Ethical approval was obtained from the Bioethical Committee of CSIC and from the Regional Ethics Committee for Clinical Research of the Principality of Asturias in compliance with the Declaration of Helsinki of 1964, last revised in 2013. All the volunteers, or their legal representatives, gave written informed consent. The exclusion criteria considered for this study were not being diagnosed of autoimmune diseases and not having consumed any antibiotics or probiotics during the previous month of recruitment. All measurements were carried out in accordance with approved guidelines and regulations.

**TABLE 1:** Fecal short-chain fatty acid (SCFA), branched short-chain fatty acids (BCFA), and total SCFA/BCFA ratio, as well as general characteristics and daily dietary intakes of the studied population according to age groups.

	Babies	18–50 years	51–65 years	66–95 years
*N* individuals	65	61	69	37
Mean age (years)	0.25	38.95 (33.5–44)	57.93(55–61)	80.32(77–85.5)
Isobutyrate (mM)	0.48^a^ (0.00–1.01)	1.68^b^ (1.11–2.12)	1.80^b^ (1.08–2.32)	1.96^b^ (1.09–2.21)
Isovalerate (mM)	0.64^a^ (0–0.52)	2.43^b^ (1.48–3.02)	2.65^b^ (1.50–3.53)	2.57^b^ (1.39–3.17)
BCFA (mM)	1.12^a^ (0.00–1.52)	4.12^b^ (2.57–5.05)	4.45^b^ (2.57–6.01)	4.53^b^ (2.49–5.28)
Total SCFA (mM)	108.51^c^ (60.96–131.77)	87.40^c^ (63.72–105.30)	71.59^b^ (46.53–87.32)	47.71^a^ (22.76–59.97)
Total SCFA/BCFA (mM)	158.01^d^ (38.28–224.86)	28.07^c^ (12.56–37.94)	20.65^b^ (11.07–23.73)	12.19^a^ (6.39–15.70)
BMI (kg/m^2^)	–	32.05 (24.99–40.82)	28.97 (23.68–30.06)	28.36 (26.03–30.96)
Energy (kcal/day)	–	1934.50^b^ (1577.03–2160.61)	1967.27^b^ (1601.67–2207.05)	1715.07^a^ (1399.65–1983.15)
% carbohydrate	–	42.68 (38.08–29.82)	41.34 (36.01–47.38)	40.73 (36.40–44.92)
% protein	–	19.03 (15.88–21.11)	19.27 (16.13–21.76)	19.46 (17.82–21.52)
% fat	–	36.69 (29.82–40.93)	36.74 (32.23–40.56)	38.82 (35.44–41.73)
Fiber (g/day)	–	18.49^ab^ (13.09–22.01)	22.73^b^ (17.80–27.70)	16.65^a^ (12.47–20.34)
Insoluble fiber (g/day)	–	12.43^b^ (13.09–22.02)	14.09^*c*^ (10.43–19.20)	10.12^a^ (1.61–2.74)
Soluble fiber (g/day)	–	2.33^b^ (1.60–2.56)	3.03^*c*^ (2.20–3.74)	2.15^a^ (1.61–2.74)

*Results are presented as mean values; between parentheses, first and third quartile values for each variable within each group are indicated. BCFA: isobutyrate + isovalerate. Total SCFA: acetate + propionate + butyrate + caproate + valerate + isobutyrate + isovalerate. Different lowercase letters indicate significant differences among groups of age for the variable considered (p < 0.05).*

**TABLE 2:** Fecal short-chain fatty acid (SCFA) levels, branched short-chain fatty acids (BCFA), and total SCFA/BCFA ratio, as well as general characteristics and daily dietary intakes of the studied population along the different BMI groups.

	BMI < 25	BMI 25–29.9	BMI 30–39.9	BMI ≥ 40
*N* individuals	40	63	27	25
BMI (kg/m^2^)	22.24 (20.91–23.48)	27.34 (16.17–28.40)	33.61 (31.14–35.78)	44.89 (41.55–46.10)
Isobutyrate (mM)	1.56^a^ (0.98–1.81)	1.79^a^ (1.04–2.13)	1.74^a^ (1.02–2.19)	2.11^b^ (1.55–2.69)
Isovalerate (mM)	2.10^a^ (1.21–2.36)	2.51^a^ (1.39–3.20)	2.54^a^ (1.34–3.54)	3.15^b^ (2.43–4.03)
BCFA (mM)	3.67^a^ (2.31–4.03)	4.30^a^ (2.33–5.33)	4.28^a^ (2.42–5.53)	5.26^b^ (4.15–6.65)
Total SCFA (mM)	65.86^a^ (46.47–85.38)	64.93^a^ (33.37–90.49)	75.16^ab^ (47.55–98.26)	95.80^b^ (53.83–130.49)
Total SCFA/BCFA	22.04 (12.46–28.42)	20.60 (9.75–21.63)	23.89 (9.70–32.98)	22.11 (10.47–29.73)
Energy (kcal/day)	1883.32^ab^ (1557.79–2133.54)	1813.90^a^ (1.497–2.051)	2152.32^b^ (1897.44–2380.26)	1832.71^ab^ (1478.64–2142.19)
% carbohydrate	41.05 (35.00–46.03)	41.90 (37.57–48.13)	42.76 (36.84–48.32)	39.54 (31.90–46.64)
% protein	18.49^a^ (15.96–21.50)	19.21^a^ (16.47–21.44)	18.32^a^ (15.72–20.35)	22.32^b^ (18.46–26.25)
% fat	37.70 (32.89–42.41)	37.34 (33.83–40.77)	36.99 (30.41–40.89)	36.81 (28.83–43.09)
Fiber (g/day)	20.27 (12.83–23.58)	19.04 (14.40–23.11)	21.05 (14.38–26.71)	20.05 (13.32–25.31)
Insoluble fiber (g/day)	13.09 (8.17–17.66)	11.95 (8.61–14.41)	14.37 (8.96–18.85)	14.13 (10.05–18.52)

*Results are presented as mean values; between parentheses, first and third quartile values for each variable within each group are indicated. BCFA: isobutyrate + isovalerate. Total SCFA: acetate + propionate + butyrate + caproate + valerate + isobutyrate + isovalerate. Different lowercase letters indicate significant differences among BMI groups for the variable considered (p < 0.05).*

### Nutrition Assessment and Anthropometric Evaluation

Dietary information was registered by means of an individual interview, of approximately 1 h, through an annual food frequency questionnaire (FFQ) of semi-quantitative type, composed of 160 different foods items. Interviewers were previously trained to standardized dietary assessment. Methodological issues concerning interview process and portion size quantification have been previously published elsewhere (Fernandez-Navarro et al., 2017). Conversion to nutrients was developed by using information available from the Center for Higher Education in Nutrition and Dietetics (CESNID), in energy, macronutrients, vitamins, and minerals [Centro de Enseñanza Superior de Nutrición Humana Y Dietética (CESNID), 2008]. To detail the type of protein consumed (animal or vegetal origin), the food composition tables published by the United States Department of Agriculture (USDA) were used (Fernández-Navarro et al., 2018).

Size and weight were determined in adults, in order to calculate BMI, by dividing the weight (kg) by the square of height (m^2^). A dietary caloric profile was obtained individually, by calculating the contribution of each of the macronutrients to the total daily energy intake.

### Fecal Collection and Metabolite Analysis

Fecal sample collection was performed as indicated in previous studies (Arboleya et al., 2012, 2016; Salazar et al., 2013; Fernandez-Navarro et al., 2017). In brief, fecal material was collected in sterile containers, immediately frozen at −20°C, and transported to the laboratory. In the laboratory, 1 g of fecal samples was diluted 1/10 in phosphate-buffered saline solution (PBS) and homogenized in a LabBlender 400 stomacher (Seward Medical, London, United Kingdom). Supernatants were obtained by centrifugation (10,000 rpm 10 min, 4°C), filtered through 0.2-μm filters, added with 1/10 2-ethyl butyric acid (1 mg/mL) as an internal standard and stored at −80°C until analysis. SCFA were identified and quantified in a gas chromatograph 6890N (Agilent Technologies Inc., Palo Alto, CA, United States) connected to a mass spectrometry 5973N detector (Agilent) and to a flame ionization detector (FID), and data were collected through an Enhanced ChemStation G1701DA software (Agilent) as described previously (Fernández-Navarro et al., 2018). SCFA (acetic, propionic, butyric, isobutyric, isovaleric, caproic, and valeric acids) were identified by comparison of their mass spectra with those held in the HP-Wiley 138 library (Agilent) and by comparing their retention times with those of the corresponding standards (Sigma-Aldrich, St. Louis, MO, United States). The peaks were quantified as relative abundances with respect to the internal standard. The absolute concentration (mM) of each compound was calculated through linear regression equations (*R*^2^ ≥ 0.99) from the corresponding standard curves obtained with different concentrations. Molar proportions (MP) were also determined by referring the concentration of each of the SCFA to the sum of concentrations of all SCFA considered this as 100%. In the same way, MP of BCFA (isobutyric + isovaleric acids) was determined by referring them to the sum of concentrations of all SCFA.

### Statistical Analysis

Statistical analyses of results were carried out using IBM SPSS version 22.0 (IBM SPSS, Inc., Chicago, IL, United States). Data for the different variables analyzed were compared among the different groups of age as well as among adults with different BMI. The Kolmogorov–Smirnov test (K–S) with Lilliefors significance correction indicated that most variables did not show a normal distribution. When the distribution of variables was skewed, the natural logarithm of each value was used in the statistical test. Mean values are presented as untransformed variables. The absence of collinearity between age, BMI, and sex was corroborated for each BCFA. With this collinearity being negative, analyses for age were performed in all samples whereas BMI statistics were only performed in adults because this parameter is not applicable in babies. The Kruskal–Wallis test for pairwise comparisons with Bonferroni correction was then used to assess all statistical comparisons between groups. SCFA molarities were used to perform a principal component analysis (PCA) of SCFA production through life and across different BMIs in adults with SPSS, whereas graphical plots were constructed with plot.ly package for RStudio version 1.1.463. A Spearman correlation analysis was also conducted to investigate the associations between BCFA and anthropometric and dietary factors in the study population.

## Results

### Fecal Branched Short-Chain Fatty Acids Along Life

**FIGURE 1:**
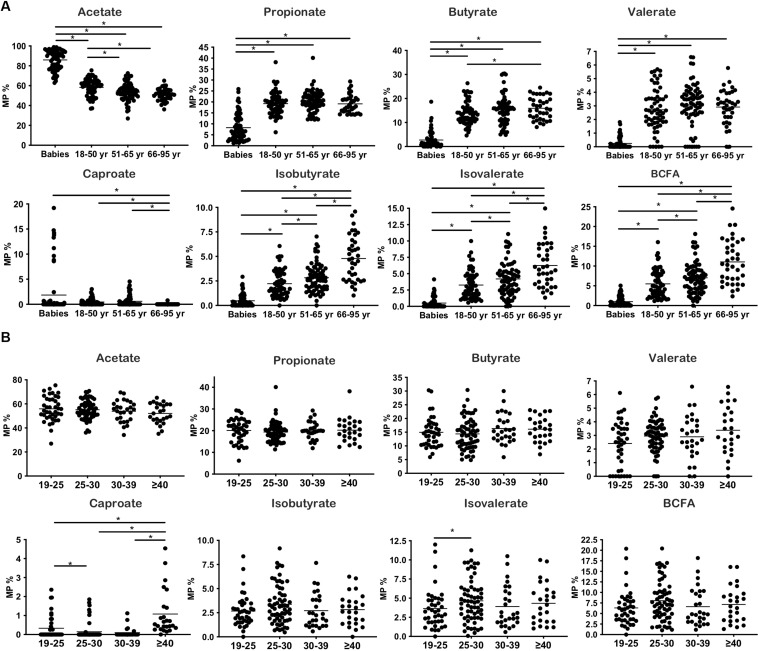
Changes in SCFA production throughout life and BMI increases. **(A)** Molar proportions of SCFA during aging. **(B)** Molar proportions of SCFA throughout BMI increases. *p < 0.05.

**FIGURE 2:**
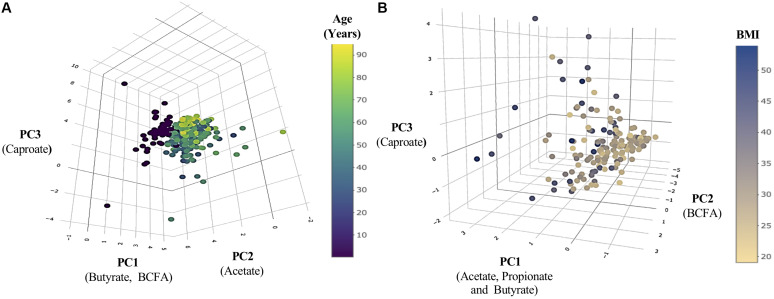
Population distribution regarding changes in SCFA production throughout life and BMI increases. **(A)** Principal component analysis (PCA) of SCFA production throughout life. **(B)** PCA of SCFA production according with the increase of BMI. The main components of each axis are indicated below them in parentheses.

The general characteristics of the studied population, according to the age groups analyzed, are presented in Table 1. The fecal levels of total SCFA were significantly lower in adults than in pre-weaned infants whereas in contrast BCFA concentrations were significantly (*p* < 0.05) lower in babies than in adults. Total SCFA in adults decreased with age (*p* < 0.05) while BCFA fecal levels were stable among the adult age groups. As a consequence, the total SCFA/BCFA ratio showed a decline with age (*p* < 0.05), mainly due to a significant decrease in SCFA fecal levels, specially acetate and propionate (Supplementary Table S1). In order to minimize the bias that variations in water content of the different fecal samples could introduce on statistical comparisons of fecal concentrations of BCFA, MP of each of the different SCFA were also calculated and compared (Figure 1A). This way, MP of isobutyric and isovaleric acids and total BCFA evidenced a significant increase throughout aging (*p* < 0.05) (Figure 1A). Regarding major SCFA, the MP of acetate showed clearly higher fecal levels in infants than in adults, with fecal concentrations of this compound showing a significant decrease through age until the 51–65 year-old group. In contrast, infants displayed significantly lower fecal MP of propionic, butyric, and valeric acids than did adults (Figure 1A). A PCA was then performed with the concentrations (mM) of the different SCFA for the whole population under study. The three components resulting from the PCA explained 85.64% of the variance (PC1 18.18%, PC2 21.49%, and PC3 15.96%). BCFA and butyrate were the main components in PC1 (Figure 2A), acetate in PC2, and caproate in PC3. Fecal samples of babies had a clear different distribution than those of adults, and among adults, a gradient along PC2 can be appreciated.

Anthropometric and dietary analyses were carried out in adults (Table 1). Differences in BMI among age groups were not significant (*p* > 0.05). However, a significant (*p* < 0.05) reduction in energy intake was evidenced in the group of 66–95 years (Table 1), which resulted in lower intakes of carbohydrates and protein (data not shown); in spite of this, the contribution percentage of these macronutrients to the total caloric intake remained without variations (Table 1). Fiber consumption, soluble and insoluble, was significantly lower in elderly people than in the other two adult groups (*p* < 0.05) whereas the group of 55–65 years presented the highest consumption.

### Fecal Branched Short-Chain Fatty Acids as Related With BMI

The general characteristics of the studied population according to BMI are presented in Table 2. Babies were not considered in analyses related to BMI, because this parameter is accurate only in adults. Subjects with morbid obesity (BMI ≥ 40) displayed significantly higher concentrations (*p* < 0.05) of total SCFA and BCFA in feces than the other weight groups: normal-weight (BMI 18.5–24.9), overweight (BMI 25–29.9), and obese (BMI 30–39.9) volunteers. MP of acetate, propionate, butyrate, valerate, and isobutyrate and the sum of BCFA (isobutyric + isovaleric acids) remained without significant variations along BMI groups (Figure 1B). In contrast, MP of caproate increased significantly in morbid subjects whereas lower proportions of isovalerate were found in normal-weight people than in overweight individuals.

As with regard to the dietary information, a higher intake of calories (Table 2), carbohydrates, and fat (g/day) was evidenced in the obese group than in normal-weight or morbid obese subjects (Supplementary Table S2). Underreporting of energy and protein intake has been shown with the increase in BMI with different diet assessment methods, including FFQ (Stice et al., 2015; Trijsburg et al., 2017). However, when comparing the caloric dietary contribution percentage of each macronutrient, the only difference observed was in the proportion of calories resulting from dietary protein, which was significantly higher (*p* < 0.05) in the morbid obese group.

Principal component analysis (Figure 2B) was performed based on the adult cohort data, and the three resulting components explained 87% of the variance (PC1 53.59%, PC2 22.57%, and PC3 11.26%). The main contributors to PC1 were acetic, propionic, and butyric acids (Figure 2B), to PC2 BCFA, and caproic acid to PC3. A clear discrimination among groups was not obtained in the case of BMI.

### Correlation of BCFA Levels With Anthropometric and Dietary Parameters

**FIGURE 3:**
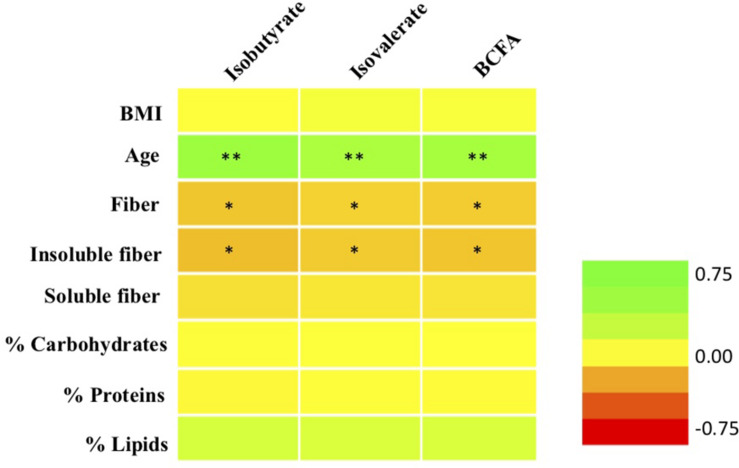
Correlations between molar proportions of BCFA (total BCFA, isobutyrate, isovalerate) with age, BMI, and daily dietary intake of macronutrients and fiber. ^∗^*p* < 0.05; ^∗∗^*p* < 0.01. Green, positive correlations; orange-red, negative correlations.

Excluding babies, correlation analyses between molar proportions of fecal isovalerate and isobutyrate and the sum of both BCFA were performed with all the anthropometric and dietary parameters studied. Significant correlations are shown in Figure 3. Isovalerate and isobutyrate levels correlated positively between them (data not shown) as described by previous authors (Cardona et al., 2005) and both of them with age (*p* < 0.01). BCFA production was not significantly correlated with fat, protein, either animal or vegetal, or carbohydrate intake (Figure 3). A less strong but negative association (*p* < 0.05) was found between these two compounds and fiber consumption, particularly insoluble fiber. In addition, MP of isovalerate and isobutyrate were not correlated with energy, vitamin, minerals, or amino acid intake (data not shown).

## Discussion

Short-chain fatty acids production and their relationship with host health status have been receiving a deal of attention during the last decade (Koh et al., 2016; Ríos-Covián et al., 2016), but studies about the production of BCFA and their effects in host health are still very scarce. To the best of our knowledge, this is the first report describing the evolution of BCFA production profile in a healthy human population along life, from newborns to elderly, and by comparing different BMI groups of adults.

MP of BCFA in the group of babies were lower than in adults, but in accordance with that indicated by other authors (Bridgman et al., 2017), infants displayed higher MP of acetate. Regarding adults, we found age as the variable that most clearly correlated positively with the MP of BCFA. Also, the SCFA/BCFA ratio decreased significantly from the age group of babies to the 66–95-year-old group, most likely due to a decrease in the levels of SCFA, mainly acetate and propionate. In contrast, previous studies did not report differences in the MP of BCFA between elderly subjects and middle-age adults (Salazar et al., 2013). This may be due to the inclusion in the previously cited study of only mature adults (>57 years) and not younger adults as is the case of the present work.

In the adult population, BCFA levels showed a negative correlation with fiber consumption, mainly insoluble fiber. In this regard, an intervention study has previously shown a decrease in intestinal BCFA production in healthy preadolescent children consuming wheat bran (François et al., 2014). Lower levels of BCFA have been also reported when comparing high carbohydrate (including fiber)/low protein versus high protein/low carbohydrate diets in human adults with metabolic syndrome, as well as in animals (Pieper et al., 2012; Hald et al., 2016). Therefore, one of the possible explanations for the higher MP of BCFA observed by us in the elder population could be related with a reduced availability of fermentable carbohydrates in the large intestine, due to their reduced intake with diet. Although the data obtained in this study are not comparable with those of intervention studies, we can hypothesize the existence of an inverse association between fiber intake and the consumption of vegetable proteins that would be in line with the results proposed in these works. It is important to point out that all cited studies compare the BCFA production after a dietary intervention or amino acid supplementation, whereas levels of BCFA production in feces of individuals with their usual diets have not been reported previously. Taking this into account, changes in diet might be a factor affecting the SCFA and BCFA production, but also it may be related to a physiological decline in the activity of the microbiota with age, as it has been previously reported (Salazar et al., 2019). In fact, undigested fermentable carbohydrates can be used by microbial BCFA producers such as *Bacteroides* or other colonic microorganisms. *In vitro* studies have demonstrated that when *Bacteroides fragilis* grew in slowly fermentable carbon sources, a shift in its metabolism through protein fermentation occurred (Rios-Covian et al., 2017). Some *Clostridium* and *Propionibacterium* species are also able to produce BCFA, although their carbon source preferences have not been studied in depth yet (Elsden and Hilton, 1978; Smith and Macfarlane, 1998). As BCFA production has been correlated in the present study with less fiber consumption, it may be possible that the reduced availability of fermentable carbohydrates by the intestinal microbiota promoted a shift to more protein fermentation and, as a consequence, an enhanced BCFA production in the gut. In addition, host changes associated with aging, as higher rate of apoptotic cells of aged gut, could lead to more availability of fermentable amino acids for the microbiota, which then may result in higher levels of BCFA (Moorefield et al., 2017).

In previous studies by other authors, high protein diets have been related with higher levels of BCFA (Pieper et al., 2012; Aguirre et al., 2016). In accordance with this, our group of BMI ≥ 40 showed significantly higher protein intake and higher production of SCFA, total BCFA, isobutyrate, and isovalerate, as well as higher proportions of caproate than did the other human groups with lower BMI. In spite of this, we did not find any significant correlation between the relative proportions of BCFA and BMI in the whole population of adults analyzed. These results indicate that whereas total SCFA levels seem to be affected by BMI increase, a direct relationship between BCFA and higher protein intake is not evident in our adult population. One limitation of this study is that, even though FFQ is widely used in the context of diet–microbiota relationships, underreporting of energy and protein intake has been shown with the increase in BMI using this method (Stice et al., 2015; Trijsburg et al., 2017). Adding 24-h recalls or biochemical indicators of protein intake has been reported as a way to overcoming this limitation, especially in the relationship of BCFA with BMI (Freedman et al., 2018). Further research is needed to make conclusions about the relationship of BCFA and obesity.

Production of BCFA by the gut microbiota seems to be affected by several factors that might influence the availability in the gut of fermentable amino acids, as could be diet, host endogen metabolism, and the intestinal microbial composition and the metabolic preferences of the different microbial groups. On the other hand, some literature has related the levels and MP of BCFA to some diseases such as depression, Rett syndrome, and anorexia nervosa (Mack et al., 2016; Szczesniak et al., 2016; Borghi et al., 2017), pointing to their potential as possible future biomarkers of health. Further research is needed in order to elucidate the mechanisms of action of BCFA in health and disease. In this regard, recent studies have reported a relationship between BCFA and lipid metabolism; thus, fecal levels of BCFA have been found to be higher in subjects with hypercholesterolemia in comparison with normocholesterolemic individuals, with fecal isobutyric acid levels being associated with a worse lipid profile in serum (Granado-Serrano et al., 2019). *In vitro* experiments with rat and human adipocytes had shown an inhibition of cAMP-mediated lipolysis and insulin-stimulated lipogenesis by BCFA, with isobutyric acid also potentiating insulin-stimulated glucose uptake (Heimann et al., 2016).

One of the limitations of this study is that the measurement of SCFA and BCFA production was performed in feces, which reflects the concentrations of these compounds at the end of the digestive tract, but not necessarily that of the other parts of the colon. Moreover, levels do not constitute a direct reflection of production by the intestinal microbiota since absorption processes are also implied. On the other hand, the content of water has not been considered in the calculation of SCFA and BCFA concentrations; however, the comparison of MP of the different SCFA compounds used in this study partly prevented the bias introduced by the different water content of feces from different individuals. Further studies are needed to shed light into the main factors affecting BCFA production and the hierarchy of their relationships in the human intestinal environment.

## Data Availability Statement

The datasets generated for this study are available on request to the corresponding author.

## Ethics Statement

Ethical approval was obtained from the Bioethical Committee of CSIC and Regional Ethics Committee for Clinical Research of the Principality of Asturias in compliance with the Declaration of Helsinki of 1964, last revised in 2013. All the volunteers, or their legal representatives, gave written informed consent.

## Author Contributions

SA, NS, and AN collected and processed the samples. SG performed the nutritional assessment and anthropometric evaluation. CR-G, MG, and DR-C designed the study. DR-C and SG performed the statistical analysis. DR-C wrote the manuscript. CR-G and MG helped with the manuscript review. All authors have read and approved the final version of the manuscript.

## Conflict of Interest

The authors declare that the research was conducted in the absence of any commercial or financial relationships that could be construed as a potential conflict of interest.
