# Supplementary material for: An Overview on Fecal Branched Short-Chain Fatty Acids Along Human Life and as Related With Body Mass Index: Associated Dietary and Anthropometric Factors
Source: Front Microbiol. 2020 May 27;11:973. doi: 10.3389/fmicb.2020.00973 (PMC7271748; doi:10.3389/fmicb.2020.00973)
Supplement: Supplementary file 1 [file Data_Sheet_1.docx]

**Table S1. SCFA levels (mM) according to age groups along with macronutrients intake.** Results are presented as mean values; between parentheses, first and third quartile values for each variable within each group are indicated. Different lower case letters indicate significant differences among groups of age for the variable considered (p < 0.05).

|  | **3 months** | **18-50 years** | **51-65 years** | **66-95 years** |
| --- | --- | --- | --- | --- |
| **n** | 65 | 61 | 69 | 37 |
| **Acetate** | 92.57^d^  (54.04-116.87) | 51.19^c^  (38.26-63.36) | 37.90^b^  (24.17-45.88) | 23.72^a^  (11.29-30.61) |
| **Propionate** | 8.70^a^  (2.52-11.06) | 17.09^c^  (9.64-21.36) | 14.90^b^  (8.24-17.41) | 9.61^a^  (3.55-12.90) |
| **Butyrate** | 2.91^a^  (0.59-3.52) | 12.44^c^  (7.15-14.81) | 11.76^b^  (5.15-14.80) | 8.42^b^  (3.30-11.33) |
| **Valerate** | 0.31^a^  (0.00-0.25) | 2.19^c^  (1.53-2.87) | 2.09^c^  (1.20-2.90) | 1.41^b^  (0.54-1.83) |
| **Caproate** | 2.89^b^  (0.00-0.19) | 0.39^b^  (0.00-0.45) | 0.48^b^  (0.00-0.38) | 0.02^a^  (0.00-0.00) |
| **Carbohydrates**  **(g/day)** |  | 204.96^b^  (165.33-246.86) | 203.84^b^  (163.32-236.16) | 173.15^a^  (138.92-198.15) |
| **Fat**  **(g/day)** |  | 79.71  (54.59-97.45) | 80.77  (60.20-93.22) | 74.52  (60.49-90.14) |
| **Protein**  **(g/day)** |  | 89.93^ab^  (69.17-102.34) | 92.98^b^  (78.83-109.33) | 83.36^a^  (68.36-98.15) |
| **Animal Protein**  **(g/day)** |  | 61.25  (42.74-75.17) | 61.95  (45.97-74.83) | 56.51  (42.65-69.40) |
| **Plant Protein**  **(g/day)** |  | 25.29  (17.51-30.57) | 29.25  (21.23-33.80) | 25.26  (20.40-29.43) |

**Table S2. SCFA levels (mM) according to BMI (Kg/m^2^) groups along with macronutrients intake.** Results are presented as mean values; between parentheses, first and third quartile values for each variable within each group are indicated. Different lower-case letters indicate significant differences among groups of BMI for the variable considered (p < 0.05).

|  | **<25** | **25-29.9** | **30-39.9** | **≥40** |
| --- | --- | --- | --- | --- |
| **n** | 40 | 63 | 27 | 25 |
| **Acetate** | 37.01^ab^  (24.30-52.10) | 35.50^a^  (17.67-46.07) | 40.41^ab^  (25.11-52.08) | 51.67^b^  (24.82-73.12) |
| **Propionate** | 13.17^a^  (8.13-17.24) | 13.48^a^  (6.17-17.39) | 15.61^ab^  (8.97-22.09) | 19.12^b^  (10.59-21.36) |
| **Butyrate** | 10.19^a^  (5.94-13.40) | 9.71^a^  (4.03-13.37) | 12.69^ab^  (7.59-16.54) | 16.14^b^  (7.22-21.54) |
| **Valerate** | 1.56^a^  (0.38-1.95) | 1.84^a^  (0.94-2.54) | 2.05^a^  (0.83-2.66) | 2.71^b^  (1.96-3.68) |
| **Caproate** | 0.25^b^  (0.00-0.25) | 0.12^a^  (0.00-0.00) | 0.11^a^  (0.00-0.00) | 0.91^c^  (0.31-1.42) |
| **Carbohydrates**  **(g/day)** | 198.12^a^  (157.77-228.77) | 189.18^a^  (147.66-220.00) | 227.61^b^  (169.57-260.82) | 180.24^a^  (152.32-214.91) |
| **Fat**  **(g/day)** | 80.55^a^  (55.32-93.77) | 75.94^a^  (59.68-90.98) | 88.66^b^  (79.26-99.26) | 76.19^a^  (60.06-89.34) |
| **Protein**  **(g/day)** | 87.52^a^  (70.34-96.73) | 85.69^a^  (68.45-99.13) | 97.13^ab^  (71.14-105.87) | 100.41^b^  (89.26-111.46) |
| **Animal Protein**  **(g/day)** | 58.75^ab^  (44.79-69.51) | 57.40^a^  (40.38-68.20) | 63.91^ab^  (44.67-77.14) | 71.59^b^  (59.67-81.75) |
| **Plant Protein**  **(g/day)** | 25.95  (19.53-31.42) | 26.39  (20.19-30.90) | 30.39  (20.27-37.41) | 26.36  (18.36-32.15) |
